# The flipped house and the bubble. Domestic space in the time of coronavirus

**DOI:** 10.1186/s40410-022-00173-2

**Published:** 2022-11-07

**Authors:** Francesco Spanedda, Matteo Carmine Fusaro

**Affiliations:** 1 Department of Humanities and Social Sciences, University of Sassari, Via Roma 143, Sassari, Italy; 2 University of Sassari, Sassari, Italy

**Keywords:** Architectural design, Housing, Dwelling space, Sharing economy, Information capitalism, Cognitive economy, SoHo, ICT, Airbnb, Network society

## Abstract

The SARS-CoV-2 pandemic put the spatial layouts of contemporary housing to the test. During the strict lockdowns in the early phases of the outbreak and the limited, temporary restrictions of the later phases, the safeguarding of public health was inevitably enforced by confining people at home, thus severing, by reasons of force majeure, the traditional relationship between the house and the city. As a result, every household had to contain within itself all aspects of public and private life, regardless of the spatial qualities and the extension of the dwelling. The unexpected new role of the last defence line against the virus showed all the advantages and limits of contemporary housing and the need for a rethinking of some of their typical features. The aim of this article is to investigate how the residential space, as a whole and in its constituent parts, has become a fundamental element in a difficult period, managing to incorporate unexpected functions and requirements but also revealing a series of congenital weaknesses.

## Introduction: the coronavirus outbreak as a spatial threat

In 2020, the SARS-CoV-2 outbreak took the world by surprise, although the risk of global pandemics was far from unexpected.

In his Ted Talk in 2015, Bill Gates stated that the next major threat would come from epidemics, not from nuclear warfare (Gates 2015) as commonly perceived.

According to Gates, the clear recognition of the danger related to the deployment of atomic weapons ultimately led to huge investments in nuclear deterrence. The epidemiological risk, instead, has been largely overlooked with little to no funding for pandemic prevention. Drawing a parallel between the consequences of a nuclear war and those of an epidemic, the greatest hazard, in keeping with Gates, might come above all from virus spread: “not missiles, but microbes.”

Recent events proved Gates right: the menace represented by COVID-19 caught the world population totally unprepared.

In addition to Gates’ observations about the difference in resources allocated to the two sectors, it is worth noting that the emphasis on the risks coming from technology over those coming from nature parallels a common misconception of Modernity. Nature is supposed to be steady and tame, a still background to fast and unpredictable technological development. The same recurring hypotheses about the artificial origin of the virus seem to be a reassuring attempt to confirm that only technology is capable of forward leaps. This misconception probably contributed to the underestimation of the epidemic threat.

On the contrary, the pandemic outbreak might just be an episode in the contemporary dialectic between the natural environment and the artificial one. A dialectic that currently becomes a form of coevolution in which human behaviour is heavily influenced by the evolution of natural systems, transformed in turn by human actions, as exemplified by climate change.

In fact, researchers mostly agree^1^ about the natural origin of the virus (Andersen et al. 2020), but it is also clear that the current way of life favoured its quick diffusion, because of the high frequency of exchanges and of the great mobility of the world population in the era of globalisation (Zimmermann et al. 2020).

The high interconnectivity, which is the hallmark of these times, drastically speeds up this process of reciprocal adaptation.

The answer to the sudden outbreak had to be equally brisk and sudden, and the only way to speed it down was a severe reduction of any physical connection.

Perceived as a radical social change, the sudden avoidance of any physical contact had to be enforced by proxemics, regulating the movement of bodies through space.

The information campaigns, orchestrated all over the world to curb the spread of the epidemic, drew attention to changes in social behaviour that were fundamentally related to spatial concepts like “distancing”,^2^ “confinement,” and “fencing” of particularly sensitive areas.

While in the past, steady adjustments in the built environment could balance the slowly occurring changes in geography and climate, in this case, the alteration happened as a sudden crisis, requiring a prompt response with no time for a proper modification of the physical environment. Therefore, the first wave of pandemics had to be fought at home, the most private space in most human societies.

In this retreat from public life, the domestic space had to absorb most of the shock caused by the containment measures.

Homes played a key role in containing the first wave of the infection.

It is therefore interesting to have a closer look at how the relationship between the domestic space and its uses evolved during the health crisis, and how a kind of “domestic resilience” developed almost spontaneously.

As always, crises reveal the functioning of a system, its hidden elements, and its implicit tendencies. This paper describes how much the experience of the lock-down, and more generally the emergence of the pandemic, disclosed the mechanisms, relationships, and qualities of the domestic space in the Western world. The significance of this description lies not only in the collection of experiences, but also in the chance to look differently at some emerging trends in the design of the domestic space, which, although not specifically designed to deal with epidemics, could help in formulating spatially rich interpretations of the requirements, character, and possibilities of homes in the contemporary world. These requirements, from now on, also include the capability to face extreme situations like a pandemic outbreak.

This article also investigates a number of case studies to better understand the consequences and the emerging opportunities for architectural discourse. Some of them are actual proposals developed by architects under the pressure of the restrictions during the lock-downs. Others are unrelated to the pandemic and conceived before it. Although they do not specifically address the issues brought to the fore by the outbreak, their spatial articulation seems to offer a convincing and sound contribution, in architectural terms, to these topics. They avoid the hasty simplification dictated by the emergency and exploit the positive ambiguity and the plural meanings of the architectural form, suggesting spatial structures that can be easily adapted to incorporate the lessons in the design of homes learnt during the quarantine.

## The dwelling as a place of social and physical connections

The domestic space, generally called “home,” is a complex accretion of elements working on different registers. In fact, the same word indicates both the combination of family, personal, and affective relationships that are established inside the house, as well as its physical structure and its spatial organisation, which could be properly designed as “accommodation” or “dwelling” (Coolen and Meesters 2012).

The system of relationships involving domestic space develops on various scales. Traditionally, the house is a permeable system (Meloni 2014, p. 423) linking two radically different dimensions. On the one hand, it unfolds inwards, building a familiar and intimate enclosure. On the other hand, it opens to the outside, giving room to a series of functional, environmental, and social relationships. The domestic space is therefore structured by spatial devices designed to articulate the many ways in which these two dimensions blend. From the sequences of atriums, courtyards, stairs, elevators, galleries, and landings, through the concatenation of entrances, living rooms, and corridors, to the position of doors and windows, every element of the house mediates between the individuals and their social and environmental context.

**Fig. 1:**
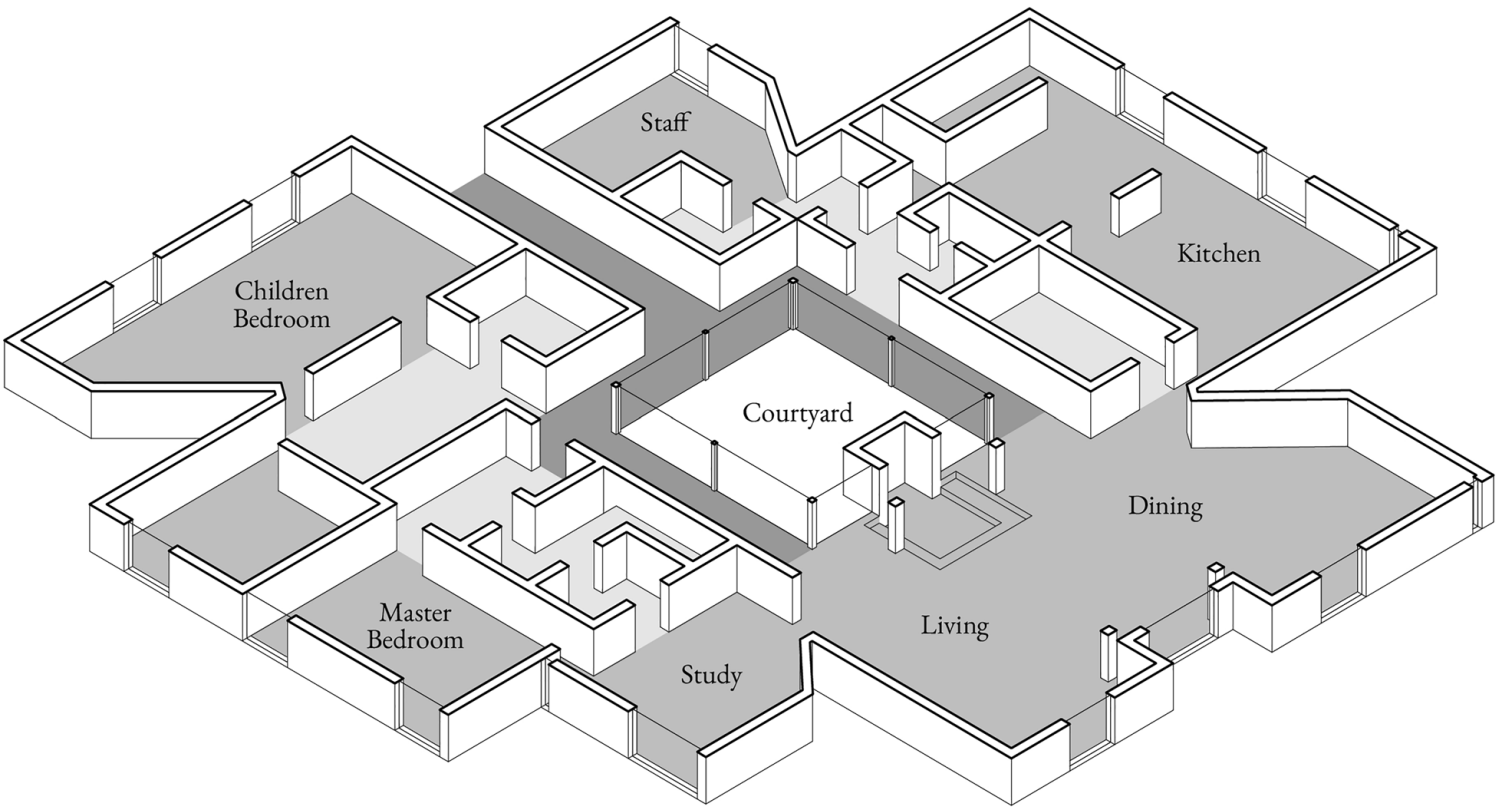
*Goldenberg House*, L. I. Kahn, 1959 (unbuilt). Dark gray: connections, medium gray: served space, light gray: servant space

The specific nature of each part led Louis Kahn to describe buildings as a “society of rooms” (Kahn 1973), a definition that perfectly fits his houses, like the *Goldenberg House* (1959), where each element is conceived as a different environment (Fig. 1).

This role, like a physical hinge between intimacy and public life, between the room and the city, is further echoed in the digital dimension. The home is in fact the place of greatest use of digital media and therefore a hub in the production and consumption of images and videos, which establishes the new system of Maussian alliances (Meloni 2014, p. 436–437) at the basis of social networking and even of new forms of creative work.

In spite of the evolving complexity of lifestyles over time, the dwelling, the physical shell of this tangled system of relationships, still largely adopts layouts whose origins date back to the deterministic legacy of functionalism (Kahler 2002), particularly the introduction of Taylorist principles in architectural design. Marked by a division between living and sleeping areas, the spatial organisation of the large majority of post-war dwellings carries on with a concept of domestic space devoted to rest and family care, reflecting the typical division of time and space of the late Second Machine Age. In this view, all the activities unrelated to family, like work and leisure, take place in different, dedicated spaces (Aureli and Tattàra 2015). The industrial concepts of function, optimisation, and minimisation, originally translated into architectural terms to offer effective and affordable accommodation to the working class, helped to maximise the exploitation of real estate. The result is a capitalist iteration of the Existenzminimum built in accordance with the functionalist principles of Tayloristic optimisation, the *deterministc dwelling*.

This spatial organisation still persists, even if habits have evolved in accordance with new forms of work, of use of public space, and because of the effects of the digital revolution (Holliss 2015).

On the other end of the spectrum is the openness of the *loft*, a de-functionalised industrial space, which glorifies the broad spaces of modernity to host a contemporary, fluid post-industrial way of life. The resulting layouts envision a unique, large space that becomes the vital core of the house by eliminating a large portion of the partition walls commonly used to divide the rooms and serving spaces.

As an alternative to the tightly tailored spaces of the *deterministic dwelling*, the open plan of the *loft* became very popular in the design of domestic environments. However, the reasons for the *loft* mainly lay in real estate strategies aimed at exploiting the old, dismissed industrial buildings, fostering their sense of spaciousness. The *loft* immediately took a foothold in the real estate market, especially among those individuals and families seeking a domestic space able to express a feeling of freedom and creativity beyond its sheer functionality, and often a refusal of the separation of work and life.

The lack, or strong reduction, of inner partitions seems antithetical to the strict functional subdivision of the *deterministic dwelling*. In fact, the coexistence of several functions and the reduced privacy in the open space of the loft are basically possible because of the low number of its occupants, and due to several social activities typically taking place in the public realm.

The amount of hours spent outside the house for work and leisure, the high real estate prices, together with the emphasis on optimisation, or the direct derivation from industrial spaces, led to a general simplification of floor plans, with the progressive strong reduction of balconies, roof-spaces, and courtyards, whose contribution to the quality of life inside the dwelling seemed irrelevant. These ancillary spaces became just accessories to raise the value of residential real estate, since social activities, including sports, body care, eating and cooking, often took place outside the house in the urban space, thus reinforcing the multiple links between the dwelling and the city.

## Quarantine and isolation

The lock-down discarded the Modernist legacy of the organisation of time and urban space around the three pillars of home, workplace, and leisure, a legacy which has been already undermined by the “end of work” (Rifkin and Canton 2002) and the new social dimensions introduced by new technologies.

During the anti-Covid seclusion, homes were suddenly called upon to perform tasks which were radically different from those they were designed for.

To be precise, the Taylorist assumption that every space should match a function gained considerable momentum among Modern architects, but not all of them agreed on it. Mies van der Rohe (Kahler 2002) and Taut (1924), clearly stated that there is no univocal correlation between the home as a place of domestic affections and its spatial structure. In his text *Die Frau als Schöpferin*, Taut openly refused the functionalist approach derived from Taylorism and described the interrelationship between rooms and activities as highly subjective, and evolving over time in a succession of interactions. The imperfect correspondence between container and content leaves room for adaptations and shifts in use.

This is much more evident in dwellings designed before the diffusion of functionalism, and is possibly one of the features that most contributed to giving homes the necessary resilience to become the last-ditch defence during lock-downs.

The first effect of the lock-down was perhaps to sever the link between the house and the city, one of the fundamental and recurring relationships within the urban environment. This connection vanished when streets and squares emptied, and families lost contact with their friends and their neighbours. As soon as the collective value of common spaces vanished because of social distancing, all the filters between inside and outside became impervious, and even the most urban and open of houses turned into fortresses.

Therefore, each family had to face the situation through temporary and emergency measures, in part by removing from everyday life everything that could not be reabsorbed within the physical space of the dwelling, in part by transforming the use of already existing parts of the house, which was turned upside down to incorporate some characteristics of the external space, in particular some of the social activities that typically took place in the public and collective spaces within the city.

While streets and squares, schools and offices became a no-man’s land where people looked for isolation and privacy, the house turned into a social place.

Both the *deterministic dwelling* and the *loft* proved to be ineffective in the new situation. The constrained space of the former was incapable of offering the flexibility and the bigger surfaces required during the lock-down, often lacking external spaces, which were considered optional when the dwellings were designed. The fluid space of the latter, instead, lacked the separation in the form of the visual and acoustic partitions needed to carry out different tasks in the same household, and to let public and private coexist.

Moreover, both spatial organisations were based on the quality of their inner relationships, while the need to think anew about the connection between outside and inside shifted the attention to the relationship between inside and outside, in order to limit the interactions between the household and the exterior, perceived as a dangerous place.

Thus, the spread of the virus strongly influenced the way in which people work, live, interact, and ultimately stay at home.

Lock-downs brought hygienic issues, like air circulation, biophilic elements, natural light, and careful selection of building materials, to the fore (Garofalo 2020). However, housing is more than hygiene and bioclimatic control. It is one of the fundamental elements of the urban settlement. Therefore, these issues should be put into a broader architectural horizon affecting spatial, functional, and semantic subjects, to preserve complexity and enrich the spatiality of the house as a contribution to the complexity and richness of the city.

This renewed interest in dwellings as complex spaces may contribute to a long-overdue review of housing design paradigms (Kahler 2002), rejecting both the determinism of the optimised space and the nondescriptness of the loft. However, the anxiety about contagion should not take the upper hand by enforcing even stricter paradigms just focused on health control.

## The bubble: shaping the isolation

As soon as the outbreak began, the health authorities in the involved countries enforced home confinement in an effort to separate the infected people from the healthy ones (Preparedness 2020). The first had to stay in a room or in a dedicated area, possibly equipped with a private bathroom and direct ventilation, to avoid direct contact with other people and their movements in common areas.

The spatial implications of these policies prompted an immediate response by architects all over the world in the first months of quarantine, from Plastique Fantastique’s *iSphere* (2019),^3^ a wearable bubble reminiscent of Haus Rucker Co’s *Environment Transformer* (1968) and Emilio Pucci’s uniforms designed for Braniff Airlines (1965), to Carlo Ratti’s *Cura* (2019),^4^ a plug-in intensive care unit (ICU) obtained from a shipping container.

Thus, the first spatial figure emerging from the coronavirus outbreak was the bubble, the definitive, possibly self-sufficient separation between individuals and between them and the urban space, until then considered as a necessary complement of the intimacy of the house, and suddenly turned into the most dangerous place, even more threatening than the wilderness.

One of the proposals most representative of these concerns is the “anti-Covid house,” or a “post-Covid house,” by Massimiliano Fuksas.^5^ Designed under the pressure of the impending threat, it mainly focuses on technical equipment like medical devices and air sanitisation systems, planimetric flexibility, minimum dimensional standards, and peculiarities drawn from the idea of cohabitation.

Like in early Modern Architecture, this proposal shapes the domestic space in accordance with medical concerns, linearly transposing hygienic rules to spatial planning. It looks like a perfect example of Colomina’s (2020) statement about the influence of our centuries-long obsession with diseases on architectural design.

**Fig. 2:**
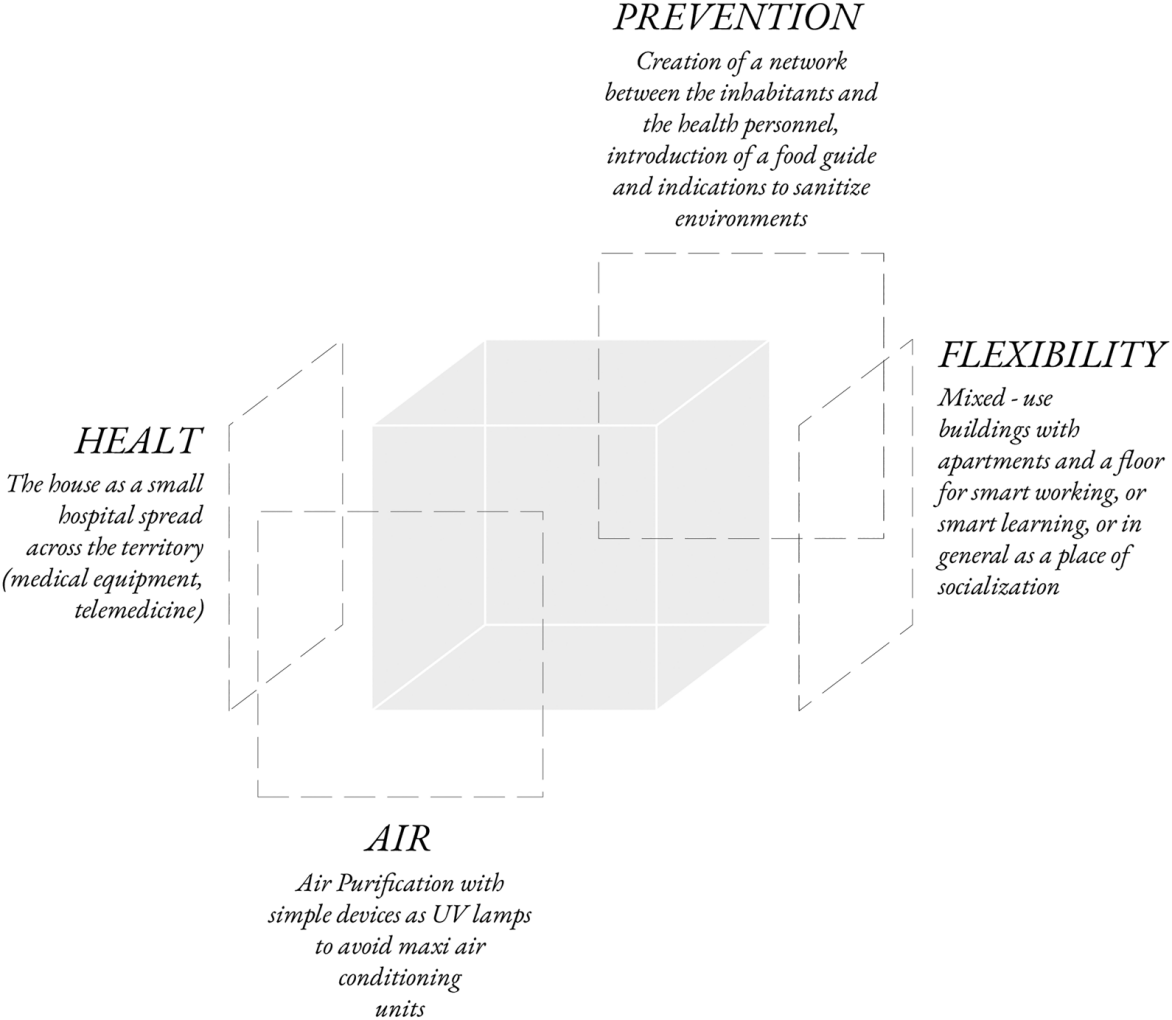
*Scheme of Post-Covid House*, M. Fuksas, 2020 (unbuilt)

The result evidently aims at building a safe shell around its inhabitants. However, while the ensuing hospitalisation and, as a result, *machinisation* of the dwelling may have been effective during earlier lock-downs, the proposal falls short of defining any relationship between the house and the public space, which, as subsequent experiences demonstrated, is critical in the case of prolonged containment (Fig. 2).

This and other comparable proposals try to deconstruct the complex topic of housing and pandemic by producing a new universal prototype whose spatiality just responds to hygienic issues. However, this approach has shortcomings. First, homes are not like cars, which are intended to be replaced with new models every few years, responding to the new requests of the market. The average number of dwellings in the OECD (2021) countries, for example, is 461 per thousand inhabitants. Since the total population of OECD members amounts to more than 1.3 billion, it is safe to estimate that no less than 59 million dwellings are in OECD countries alone. The substitution of this huge number of houses, or their upgrade to new standards, would then require a long time, longer than the development of vaccines, if not—hopefully—longer than the pandemic itself.

Second, the containment measures evolve as the infection progresses, thus discouraging an immediate translation into fixed spatial arrangements, which could become quickly obsolete. As an example, the vast majority of governments worldwide contained the first wave of infection by raising fences between the safe inner domestic space and the dangerous outer space. However, the containment of the second wave was enforced through more specific policies, with occasional local relaxations of lockdowns, while in several situations, the tables turned, and the role of houses shifted from “places of refuge” to “places of infection,” since the virus spread inside the households when people came back to schools and workplaces. While sanitary issues are without a doubt relevant, fostering complexity seems a better solution than oversimplification. Although not related to the coronavirus outbreak, several architectural examples of recent years successfully deal with the theme of the separation and independence of domestic spaces, producing a rich spatial complexity. These examples could also serve as a starting point for positively introducing these issues into discussions about post-pandemic living.

As a result, Mies and Taut’s observations on the importance of spatiality prove to be good and useful advice for designers in times of pandemic.

An interesting solution that mingles opportunities of isolation and a sense of community, both at the figural and at the pragmatic level, is MVRDV’s rooftop extension for the existing house and atelier of the Didden family in Rotterdam. It appears as a cluster of rooms, separated from the main residential unit and enjoying almost complete independence from it. Actually, the designers created a little independent village on the roof of the pre-existing historic building, marked by squares and streets that connect the new independent volumes.

**Fig. 3:**
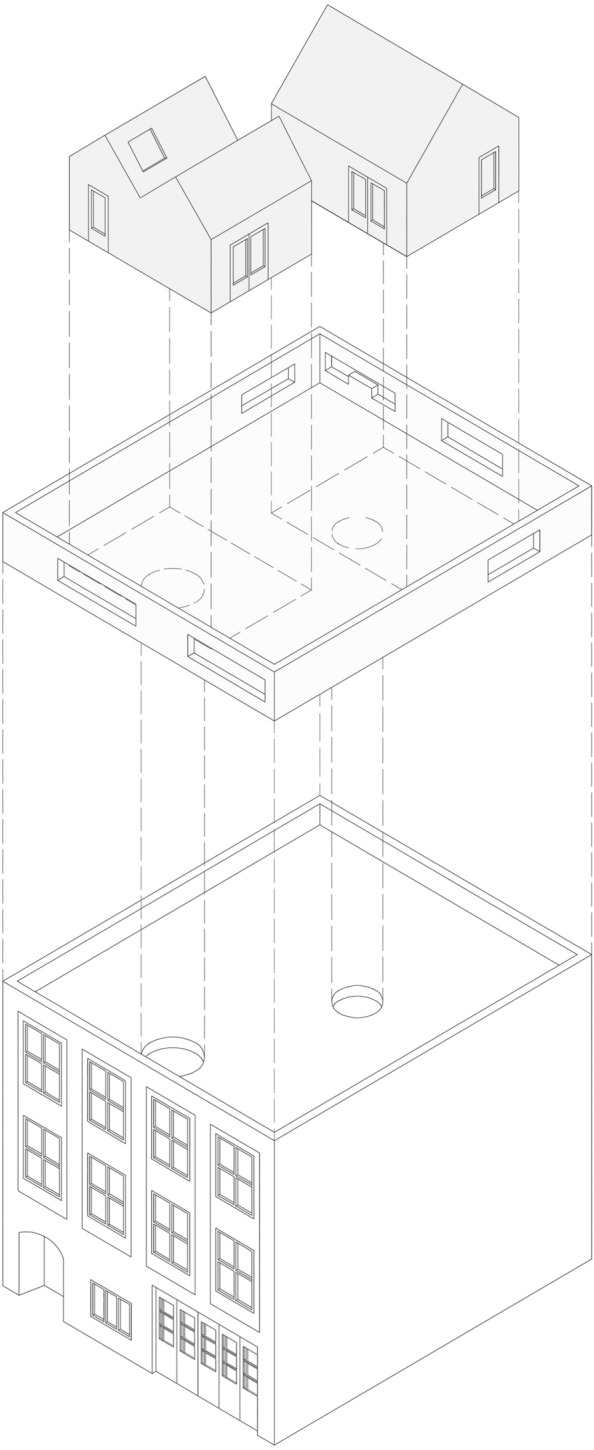
*Didden Village*, MVRDV, Rotterdam, 2006. Light gray: rooftop rooms

These new pavilions are linked to the main house through suspended spiral staircases, and they are indeed independent from the rest of the house since they host sleeping areas, a toilet, and large open living areas equipped with trees, tables, and benches. As the architects said, the rooms are conceived as separate houses, optimising the privacy of each member of the family (Fig. 3).

**Fig. 4:**
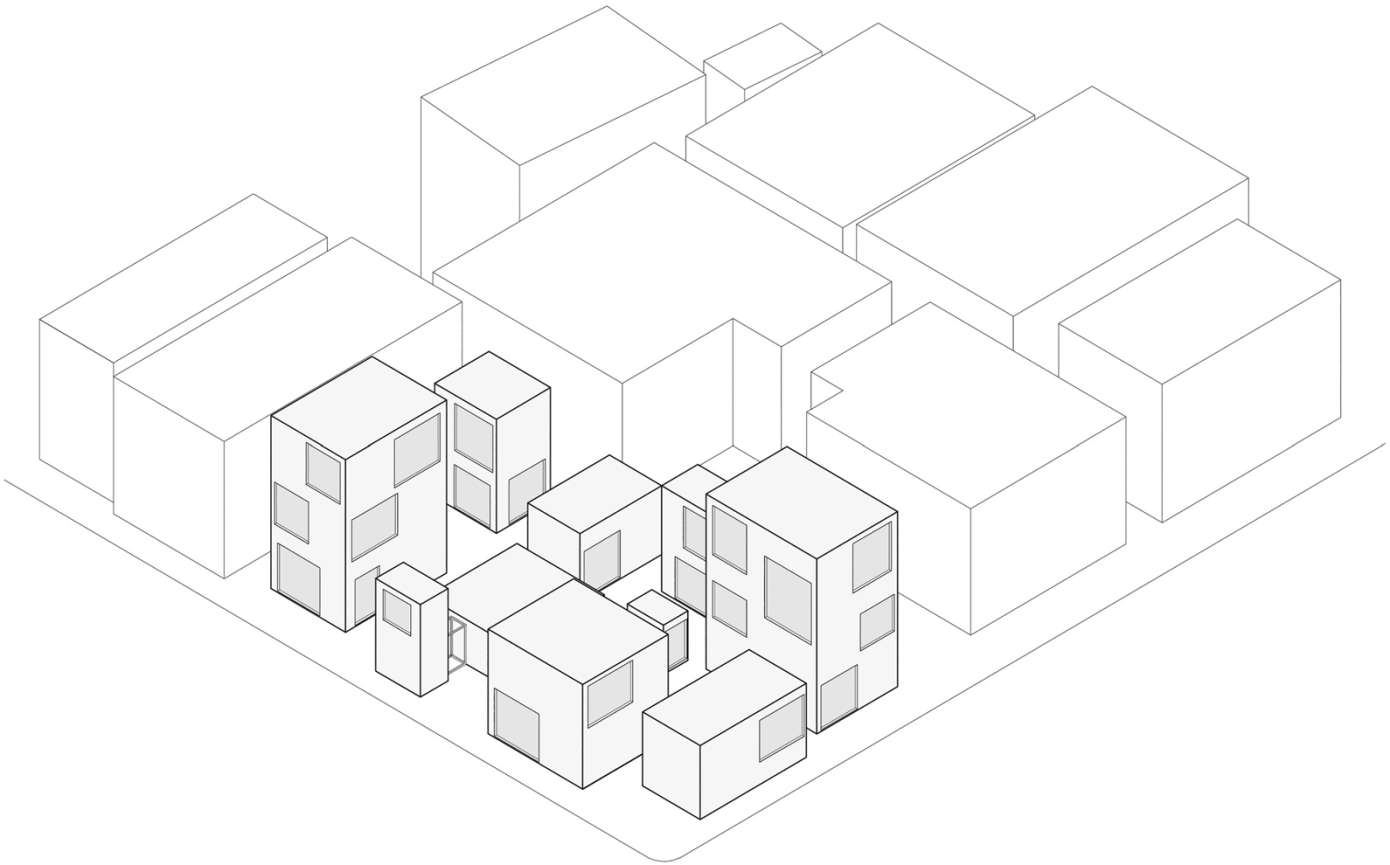
*Moriyama House*, R. Nishizawa, Tokyo, 2005. Light gray: the community of buildings

Similarly, the *Moriyama House* (2005) is a residential project by Ryue Nishizawa (founder of the Japanese office SANAA) in Tokyo, which features a “shattered” domestic space, made of detached individual rooms lying close to each other. The house consists of a dozen volumes, separate and independent buildings with different heights, connected to each other by the remaining surface of the lot, which works like a private garden, although open to public view. The house is indeed a community of buildings, all grouped in a single parcel. Thanks to its spread layout, the owners can rent the volumes that they do not use. At *Moriyama House*, domestic life takes place between inside and outside because some accessory spaces, such as toilets or showers, are placed in separate boxes. In this peculiar situation, “outside” actually means the whole neighbourhood, since no fence or boundary separates the boxes from the urban space. This spatial configuration allows for a strong, yet blurred connection between the house and the urban environment, as the urban space literally mingles with the private (Fig. 4).

Far from the dense city, the *The Distributed House* (2000),^6^ designed by OMA on Harbour Island in the Bahamas, also works on the idea of distancing and proxemics, by introducing the concept of low density at an architectural scale. Instead of stocking the house’s rooms in the vertical, this design proposal distributes the elements of the house across the jungle-covered land. Therefore, each space becomes an independent pavilion, capturing a particular feature of the landscape. The house turns then into a cluster of elements, which are the master bedroom, the guest house, a tower for children’s bedrooms, and the pool, all grouped around the dining room, the fulcrum of the space and the centre of common domestic life, described by the architects as “a mini acropolis.” This example apparently shares some common traits with the *Moryama house*. However, it unfolds its spatial organisation on a larger scale as it blends into the landscape instead of concentrating in an urban lot. But the most notable difference is the nature of the space between its elements, that in this case is completely private.

These examples show spatial organisations that feature different degrees of isolation and, at the same time, refuse the idea of severing the relationship with the outside. Instead, the assemblage of rooms itself becomes a spatial device that filters, protects, and allows for a selective relationship with the outer space. Even if not provided with medical equipment, the porous tridimensional space of the dwelling allows for air circulation, controlled movements, and interstitial spaces that could host sanitary devices without letting them take the upper hand.

## The flipped house: public goes private

When the connection between the city and the dwelling was severed, the first way to restore a social life was the “simulation of proximity”—in accordance with the successful definition of Virilio (2002)—provided by information technologies.

During the pandemic’s spread, the usage of the network infrastructure, especially where the lock-down was more severe, has undergone an unprecedented redistribution. The areas of the city served by high-speed cables, like those occupied by the tertiary sector and by educational institutions, were left empty, while the less capable networks in residential areas suffered unusual peaks due to the contemporaneity between remote work and online play of the many children left at home.

To appreciate the exceptional load on the network infrastructure of the residential areas, it is worth recalling that initiatives where game streaming platforms host events and concerts have become increasingly frequent (Tidy 2020). In addition to representing a profound change in the way of participating in public cultural life, such proposals become important catalysts to encourage the participation of people all around the world, with millions of users connected at the same time.

The sudden load on the IT network, the lack of access in sparsely populated areas, and the insufficient infrastructure in many low-income households immediately emerged as critical issues right at the beginning of the lock-downs, but there were also other issues, more related to the configuration of domestic space.

Online lessons, video conferences, and streaming amongst friends gave a collective or even public dimension to the most private rooms.

This change in the use of the domestic space was abrupt, with no time to adapt the dwelling layout. In fact, the quickest remedy against this sudden intrusion into the domestic space came via software, when almost all video call platforms offered an option to blur or replace the background.

Interestingly, the IT infrastructure in a house is mostly designed for private use and therefore more present in the most private rooms, like bedrooms.

The unprecedented and massive remote working experiment has largely been a success, leading some companies to wonder if they should ask their employees to return to their office at the end of lock-downs or whether it is actually better to keep on with remote working, as in the case of Twitter (Dwoskin 2020).

Therefore, two different strategies were deployed: on one hand, the companies offered the option to choose whether to work-from-home forever, and on the other hand, they staggered the return to the corporate buildings. The trends in the “tech” industry suggest a contrast between those who see the opportunities of agile work and those who are worried about the social aspects dictated by the disintegration of human relations between colleagues. The workers, from their point of view, have responded well to the need for “smart work,” so much so that they would positively accept its affirmation as a partial mode of employment.

Companies are therefore interested in the end of the distinction between home and workplace, on the condition that homes become places where employees can work to their full potential and without limitations. Working-from-home necessarily brings the structure of the dwelling and its features to the fore. It has to be, at the same time, a place for living, for working and for leisure, with deep semantic, constructive and organisational implications.

In addition to the erosion of privacy, the virtual expansion of the public realm had to deal with other aspects related to dwelling space. The small size of many houses (Eurostat 2020), designed to accommodate the whole family just when resting, was a reason for the interference from many online activities taking place simultaneously. Neither the functionalist dwellings, with their compressed space, nor the loft, with their lack of partitions, could provide useful separate spaces, or at least acoustically isolated niches, to allow both parents to telework while their children attend online classes.

The increasing complexity and the accumulation of activities within the living space during the lock-down brought some authors to describe their habitation in geographical terms: the thresholds between the rooms became “borders,” defining places intended for various activities, thus virtually expanding the space of the apartment within the minds of those living there (Rumiz 2020).

The overlap between domestic space and workspace has been well depicted by Beatriz Colomina (2020) through the gradually shifting role of some domestic objects over the years, suddenly boosted by the pandemic. Colomina sums up this imagery into one of the most intimate furniture in a house, the bed as a private object, which from a symbol of extreme intimacy has been transformed into a piece of public furniture, to the point of influencing our relationship with conventional public space.

The Sighvatsson House project (2005),^7^ conceived by OMA for a plot in Venice, California, represents a valid example of a spatial structure that bring to terms the need for privacy and enclosure with the openness of the loft.

The house consists of three floors, each subdivided into three spaces, summing up to nine identical rooms in shape and size. The main compositional element of the house is the external building envelope, a translucent skin that includes the serving spaces: the connections between the rooms such as stairs and corridors; the toilets; wardrobes and closets. The opening façade, the rotating kitchen, the moving shelves, the façade balcony, and the convertible room allow the inner space of the house, and the relationships within it, to change continually, morphing its spatial and functional layout and passing from an open to a discrete configuration.

Therefore, since all the interstitial spaces are located within the skin, the nine rooms build a system where the movement of each element directly affects the surrounding spaces. The partitions between the rooms are able to move, rotate, rise or open, so the house changes in accordance with the needs of the occupants based on a wide range of spatial possibilities.

## The flipped house: private goes public

Not all social activities migrated to the virtual space.

The meetings among neighbours no longer took place in the empty collective spaces like entrances, staircases, and aisles, but literally moved to the periphery of the dwelling.

Service balconies, unused flat roofs, small courtyards, and even the window thickness, that tiny border between outside and inside whose contribution to the commercial value of the building was previously deemed irrelevant, became suddenly precious. The iconography of the lock-down is crowded with people looking out, squatting in the restricted volume of the wall openings, singing together or applauding from balconies and roofs.

These peripheral places were exactly those that shrunk over time, because, in a functionalist view, they were just wasted space.

The wall thickness, for example, has been long considered something that had to be reduced to increase the inner space. However, the window stools and jambs, whose deepness, in pre-modern architecture, allowed for sitting and reading, suddenly regained their real role in establishing a connection between exterior and interior.

Similarly, the small balconies and flat roofs that hosted choirs, discussions, and even tennis matches between rooftops have been previously demoted to minor activities like laundry drying or temporary storage.

But these ancillary spaces, placed at the periphery of the building, became the places where people could enact citizenship and publicly interact, restoring the public space out of private fragments, activated in an unusual way by voices, gazes, or simple physical presence.

Thus, the horizontal public space made up of squares and streets that usually enables meet-ups through the movement and gathering of bodies across it, was momentarily replaced by a vertical public space made up of a sequence of separated niches inhabited by still bodies that allowed a form of meeting based on the crossing of looks and sounds.

This compressed and composite peripheral public space was, however, paramount in maintaining the most important requirements of urban physical space: the unexpected encounter, the serendipity, the sudden connection, through the oblique gazes between balconies and windows and terraces, between people who usually belong to different groups.

Such a feature played an important role in balancing the other side of the public space during the lockdowns: the sociality of the network, based on homogeneity, obtained through the selection of individuals and ideas through algorithms (Ratti 2020).

The health emergency brought to the fore the need to introduce, within the layout of the dwelling, exterior spaces that can at once retain a private or semi-private nature and allow for a controlled exposition to the outside. As evidenced by the increasing price of semi-detached houses in central locations (Cheshire et al. 2021), the exterior spaces became unexpectedly the most valued value during lock-downs. Thus, balconies and gardens are not just ancillary spaces or added benefits to increase the value of the house. Their absence in affordable housing cannot be replaced by public parks or collective facilities. Their function is twofold. On the one hand, they bring natural lighting and ventilation to the enclosed rooms; on the other hand, they become part of a process of extroversion of the house that becomes a hub in the manifold relationship between the residents and the world outside. One of the best examples of a retrofit that addresses this kind of issue was designed well before the coronavirus outbreak.

From this perspective, the project for the conversion of 530 housing units in the *Grand Parc in Bordeaux* (2017),^8^ designed by the French architects Lacaton and Vassal, appears to be an interesting reference.

The first aspect to notice is that Lacaton and Vassal worked on the building refurbishment of a social housing complex, showing a way to work on existing buildings and, in particular, to improve the living conditions of low-income households, which suffer from emergency situations—like lock-downs—the most.

The architects extended the domestic space by adding a further liminal space to the façade of the mass-built buildings. These winter gardens and balconies become a hinge between inside and outside and offer at the same time some extra space and proper light and ventilation.

**Fig. 5:**
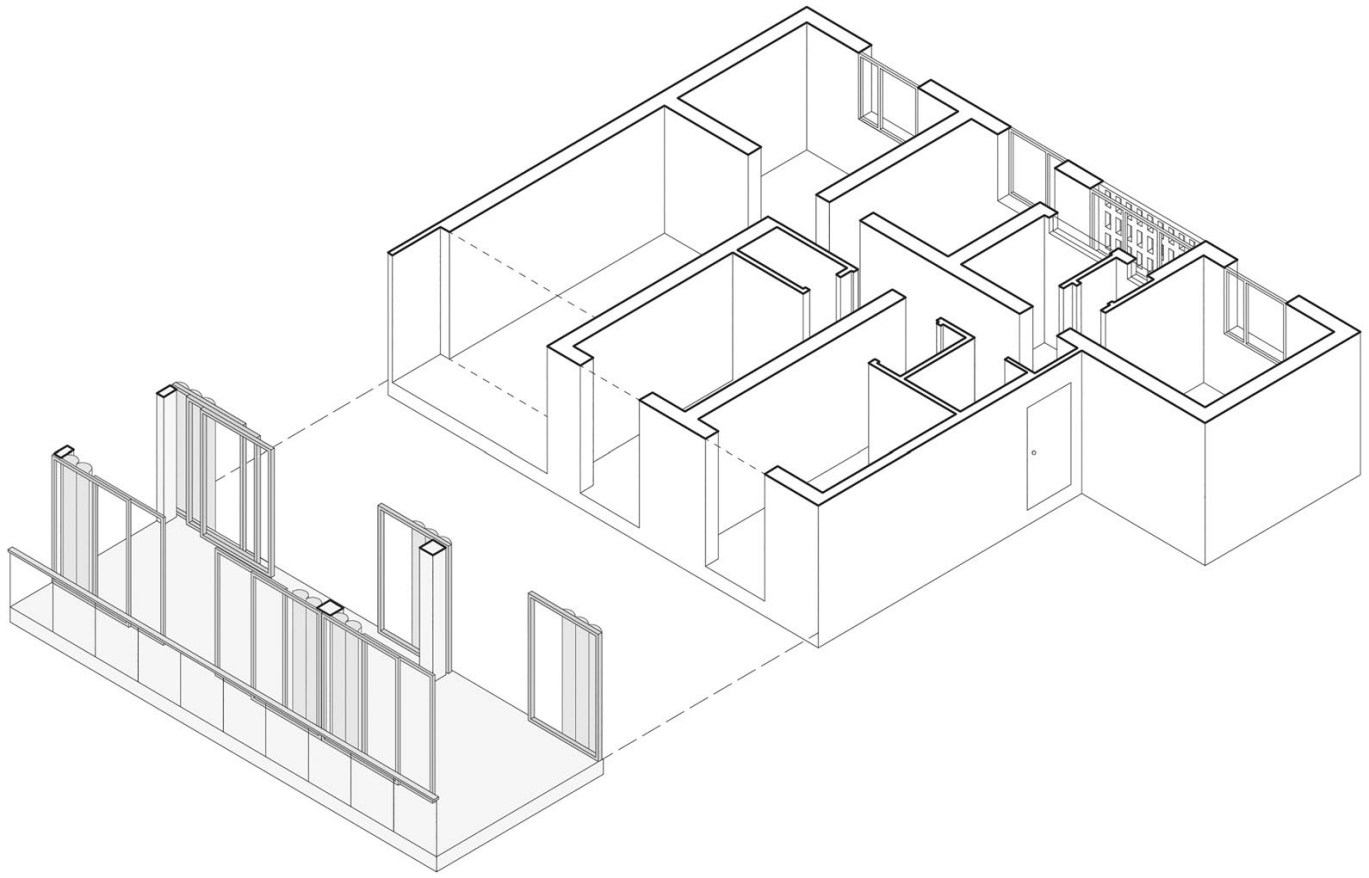
*Housing Unit in the Grand Parc*, Lacaton & Vassal, Bordeaux, 2017. Light gray: liminal space close to the façade

This addition does not affect the main structure since its structure lies side by side with the existing one, thus allowing people to stay in their homes during the building phase (Fig. 5).

The lock-down happened at the end of a time when the demand and supply of housing have found their convergence in the construction of smaller and smaller dwellings (Wilson and Boehland 2005).

But the small house, easy to buy and maintain, was immediately perceived as overcrowded during the lockdown.

The progressive reduction of the size of the house is to be found for different reasons, some of which have been questioned precisely in this period of crisis.

Certainly, the first reason is economic: buying and managing a small house is less expensive than a larger one. From 2001 to 2011, rents increased by 87% and expenses for households by up to 133%, for an overall increase in the cost of housing of 84% (Eurostat 2022). In other words, the housing conditions of the weakest groups in qualitative terms are as if they were also directly proportional to the ability to secure a large house.

A second reason is the growing importance of the “sharing economy” and its influence on the sphere of residence. The use of “hit-and-run” cities, which materialise in the form of short and medium-term hospitality, has encouraged the choice of buying a smaller house, or subdividing the larger ones built up in the “economic boom” period. The concept of “small” has therefore taken on a positive meaning. Small houses have become more attractive because their size makes it possible to use them more easily and at a more accessible cost for temporary tenants.

In Europe, the most serious housing deprivation is found in Italy, where the size of housing is clearly limited (Capolongo et al. 2020) and the overcrowding rate is 27.1% against 15.7% of the European average (Eurostat 2020).

The long time spent while constrained in small rooms emphasised the reduced size of most European dwellings (Eurostat 2020), resulting in a feeling of discomfort that turned once pleasant refuges into oppressive places.

The putting-on-display of domestic space through social media throughout the lock-down brought to the fore the deep inequality in the quantity and quality of private space available to each person, adding a new dimension to the representative function of dwellings.

Just like in the nineteenth century, the characters in Guy de Maupassant’s novels took to the streets and recognised by their clothing the people who would give them a chance to socialise. The domestic interiors—or their being disguised behind stock backgrounds—showed the interlocutor’s status.

Furthermore, many of the positive takes on quarantine as an opportunity to read, watch movies, and practise new skills also describe living situations featuring plenty of space. “Romanticizing the quarantine is a class privilege” is a slogan that appeared on a banner hanging from a balcony in Spain and was shared via Twitter (Ayala del Río 2020), pointing out how the quarantine exacerbated class differences, bringing confrontation between social groups from the public space to the interior of the house. When the size and equipment of the home become essential for survival again, they symbolise the social condition of its inhabitants again.

While many people during isolation sought to reorganise the limited space of their dwelling to turn it into a safe, but tight-fitting bubble, those with more means tried to pursue safety just by stretching the distance between themselves and the others. In proportion to their wealth, they looked for isolated houses in the woods, deserted islands, or underground bunkers, equipped with food supplies and whatever they needed to survive catastrophes and natural disasters.

Unsurprisingly, several expensive housing prototypes were sponsored during the pandemic. One above all, the *Klein Cabin*, designed by Bjarke Ingels (2019)^9^ for affluent city-dwellers who can choose where and how they want to live. Moving into the countryside, escaping to bunkers, flying to islands on private jets (Neate 2020) raises another important question. Although the house may be disconnected from the city in times of pandemic, its location, in crowded cities or isolated in remote places, strongly contributes to the perception of its safety. Consequently, when the public space dried up and all social activities moved through the digital networks inside the household, the latter can move to remote places where physical contact with others can be more easily reduced.

Theories that question the advantages of the city have a long tradition, from Howard (1898) to the present day.

During the earlier pandemic outbreak, the densely populated city core was perceived as a hotbed of infection.

Only apparently, lower population density equals higher protection against contagion.

In fact, later investigations showed lower mortality rates in more densely populated parts of the territory and higher rates in populated areas with lower densities (Hamidi et al. 2020). The reason is the more effective medical assistance in denser urban environments, but also because connectivity, not density, is relevant for the propagation of the virus.

In other words, less densely populated areas like hamlets and villages, with a higher degree of connectivity between the residents and with other settlements, have been hit more severely than denser city cores by the second wave of outbreaks.

Thus, the virus does not affect a particular urban form but any type of life based on exchange and connection.

There is therefore no reason to think that the pandemic alone can weaken the nature of urban centers.

## Conclusions

The changes induced in our lifestyle by the COVID-19 pandemic, which has rapidly spread all around the world since the first months of 2020, are not yet completely investigated. During the worldwide enforcement of lock-down as a strategy to contain the epidemiological emergency, homes played a leading role. They have been able to show some degree of resilience against such a sudden event, and that experience should contribute to further develop housing design.

People’s lives have been forced down a path that was unimaginable before, in a time marked by previously unseen levels of interrelation among individuals.

While the emergency status will not last forever, one year after the insurgence of a pandemic, it is still difficult to understand how long the limitations, the precautions, and the state of alert will persist. Moreover, there is still no clear indication if everything could revert to a “normal” state after an indefinite time or if a “new normal” will supersede the previous lifestyles, ensuing from a mix of pre-coronavirus habits, practises instilled by the quarantine experience, and conventions based on further scientific research about the nature and evolution of the virus.

While it may be too soon to draw direct conclusions from recent events, some emerging topics can contribute to a much-needed advancement in the discourse about housing design.

Firstly, the intrusion of public activities into the domestic space, be they working or leisure activities, calls for reconsidering both the traditional articulation of rooms in individual bedrooms and common living zones, and the nondescript extension of open spaces. Therefore, the dwelling becomes an assemblage of individual and common spaces related to activities that require more or less visual and acoustical separation, instead of reflecting the usual functions of sleeping, eating, and spending free time in homogeneous zones. These multiple degrees of separation can be achieved through specific federations of smaller spaces or through mobile and configurable partitioning elements. The dwellings thus drift away from complying with a “standard” to become a landscape that mirrors the creativity of their inhabitants (Taut 1924). Because conference calls and remote working have proven to be effective, time-saving, and environmentally friendly, they are likely to persist, at least in some form, and dwellings will need to adapt to their needs.

Secondly, the envelope, until now considered mainly as a representation of the status of the building inhabitants, or as the perimeter of their private property, or more recently as a means to exchange air and energy with the outside, turns into a proxemic device that facilitates the relationship between distant bodies by letting them weave a network of sights, voices, and gestures. As a consequence, the enclosing perimeter becomes a set of subspaces with their own manifold character. Without losing the opportunity to manage energy, windows, walls, and balconies could evolve into micro-spaces reminiscent of the complexity of loggias, wall niches, bow-windows, *diwans*, and many other intermediate spaces between inside and outside that enriched domestic architecture throughout history and recently fell into oblivion.

Thirdly, the restrictions showed the key role of ancillary spaces like balconies, small gardens, and courtyards. They cannot be considered anymore as wasted space devoid of any useful function, whose emptiness has to be filled and enclosed as soon as building regulations permit it. As a consequence, the house could become a combination of enclosed and open spaces, linked in different ways, cooperating for a more energy-efficient, healthier, and spatially richer domestic environment.

Although not strictly related to health issues, the case studies described in the text help in understanding how these instances can become part of an architectural discourse.

Ultimately, the aim of the paper is to show how the questions raised by the pandemic are not simply hygienic and can find some answers in architectural terms, adding further layers of complexity to the social interactions inside of the house.

## Data Availability

All data sources are cited within the text.

## Footnotes

1 Although a group of prominent scientists recently asked for a new investigation into the possibility of a laboratory accident (Bloom et al. 2021).

2 See, for example, the powerful video “Flatten the Curve” by the Ohio Department of Health https://youtu.be/o4PnSYAqQHU, retrieved 03/30/2021.

3
https://plastique-fantastique.de/isphere, retrieved 06/01/2021.

4
https://carloratti.com/project/cura/, retrieved 06/01/2021.

5 https://fuksas.com/portfolio-item/the-day-after-massimiliano-fuksas-redesigning-the-living-space-in-the-post-covid-house-interview-on-la-repubblica2020-april-19/, retrieved 06/01/2021.

6 https://www.oma.com/projects/the-distributed-house, retrieved 06/01/2021.

7
https://www.oma.com/projects/sighvatsson-house, retrieved 06/01/2021.

8 https://www.lacatonvassal.com/data/documents/20181214-165201LV_FchA4_HabitatTransfo_GRP_bd.pdf, retrieved 06/03/2021.

9
https://liveklein.com/houses/a45/, retrieved 06/05/2021.
